# Neutrophil gelatinase associated lipocalin a biomarker for bacterial‐induced pharyngeal infection—A pilot study

**DOI:** 10.1002/cre2.295

**Published:** 2020-04-26

**Authors:** Lena Walvik, Malene Kirchmann, Claus Antonio Juel Jensen, Søren Kristiansen, Lennart Friis Hansen, Michael Frantz Howitz

**Affiliations:** ^1^ Department of ENT Head & Neck Surgery Nordsjællands Hospital Hillerød Denmark; ^2^ Department of Clinical Biochemistry Nordsjællands Hospital Hillerød Denmark

**Keywords:** infection marker, neutrophil gelatinase associated lipocalin, NGAL, oral pathology, saliva

## Abstract

**Objectives:**

Neutrophil gelatinase associated lipocalin (NGAL) is secreted from activated neutrophil granulocytes and is considered an acute phase protein. The aim of this pilot study was to determine whether the NGAL concentration in saliva increases in response to a bacterial throat infection and identify pitfalls, which shall be taken into account in a protocol in a larger hypothesis testing study.

**Methods:**

Saliva samples for measurement of NGAL concentration where obtained from cases with an acute throat infection (*n* = 21) and controls (*n* = 24). Among cases, plasma NGAL, plasma CRP, and whole blood leukocytes, were measured as well.

**Results:**

There was no significant difference in NGAL saliva concentration between cases and controls overall (*p* = .31). For both cases and controls, the saliva NGAL concentration decreased significantly after cleansing the mouth with tap water (cases *p* = .01; controls *p* = .01). Among cases, a significant positive correlation between saliva NGAL concentrations before mouth cleansing and plasma CRP concentrations (*p* = .001) was observed. Blood neutrophil granulocyte count presented a nonsignificant positive correlation to saliva NGAL (*p* = .07).

**Conclusion:**

We could not demonstrate a simple association between the salivary NGAL concentration and pharyngeal bacterial infection. Furthermore, the salivary NGAL concentrations were higher among some controls than cases, suggesting that cofounders for example, periodontitis, uneven salivary dilution level, or other exogenous factors affect salivary NGAL content.

## 1 INTRODUCTION

Saliva is a complex solution containing multiple biomarkers of diagnostic interest. It is readily accessible and regularly used in diagnostics and monitoring of systemic and oral diseases.(Farnaud, Kosti, Getting, & Renshaw, 2010; Malamud, 2011) During an upper respiratory bacterial infection, pathogens such as *Streptococcus pyogenes* and *Haemophilus influenzae* can be present in saliva.(Slots & Slots, 2011) The rapid streptococcal test is widely used to detect the presence of group A streptococcus in patients with pharyngitis.(Centor, Witherspoon, Dalton, Brody, & Link, 1981) To identify other pathogens, an oral swab may be obtained for microbial identification and test for antibiotic resistance, which normally takes 2 days. Increased plasma measures of CRP and leukocytes can be helpful in distinguishing between viral and bacterial infections, but they are invasive tests and a systemic response may not correspond with a localized infection.(Bansal, Pandey, Deepa, & Asthana, 2014; Christensen, Kirkegaard, Randrup, & Klug, 2013; Peltola, Mertsola, & Ruuskanen, 2006) Following, it would be beneficial if a biomarker in saliva would serve as a proxy for a bacterial pharyngeal infection and assist the clinician in the decision on prescription of antibiotics or not. Further, saliva is both available, noninvasively, and holds the potential for a rapid diagnostic test.

Neutrophil gelatinase‐associated lipocalin (NGAL) is an antimicrobial/anti‐inflammatory iron binding protein produced by different cells, for example, neutrophil granulocytes and renal tubule cells, in response to various pathologic states (e.g., inflammation or infection).(Cai, Rubin, Han, Venge, & Xu, 2010; Kim et al., 2016; Xu et al., 1994; Xu, Pauksen, & Venge, 1995) Oral infections lead to extravasation and activation of neutrophil granulocytes.(Rijkschroeff et al., 2016) Whereas epithelial cells primarily produce a monomeric form of the lipocalin, activated neutrophil granulocytes are the main producers of the dimeric form of NGAL.(Cai et al., 2010) NGAL forms complexes with bacterial metalloproteases, depriving the pathogens iron uptake. Thus, NGAL acts bacteriostatic, supporting the innate immune system.(Flo et al., 2004)

The aim of this pilot study was to determine whether the NGAL concentration in saliva increases in response to a bacterial throat infection, ultimately with the intention of making a rapid diagnostic noninvasive test and second, to identify pitfalls, which shall be taken into account in a protocol in a larger hypothesis testing study.

## 2 METHODS

### 2.1 Cases and control subjects

We included patients ≥18 years of age, with clinical signs of acute upper respiratory infection. The patients were referred consecutively by general practitioners or private otorhinolaryngologist from a population size of 800,000 for in‐ or outpatient treatment at the Department of Ear, Nose and Throat (ENT), Head and Neck surgery, at Nordsjællands Hospital, Hillerød, Denmark.

At their first visit to the ENT department, cases underwent a clinical ENT examination, including collection of saliva, throat swabs, aspiration of pus for culture (when abscess was present), and blood samples (CRP and leukocytes). In case 13–21, an additional blood sample (amylase) and a supplementary saliva sample were collected after 5–10 s of mouth cleansing with tap water. Clinical information from all cases was collected using a standardized questionnaire filled in by the patient and the hospital clinician.

We conducted an unblinded pilot study and aimed to include 20 cases with acute upper respiratory infection and 20 healthy controls.

The controls were healthy employees at the ENT and Clinical Biochemistry Department. In controls, only saliva samples were collected. In control 13–24, an additional saliva sample was collected after 5–10 s of mouth cleansing with tap water.

### 2.2 Saliva

The saliva was collected using the Salivette® system (Sarstedt AG & Co. KG, Nürnbrecht, Germany). The Salivette tubes were spun for 5 min, and the saliva was kept frozen at −20°C before analysis. Initial experiments were carried out to determine whether the Salivette® filter did bind NGAL molecule, which was not the case (data not shown).

### 2.3 Blood

Blood (lithium heparin) was collected using the VACUETTE® specimen collection system (Greiner Bio‐One, Monroe, NC, USA).

### 2.4 NGAL

The saliva and plasma NGAL concentration was measured using the CE labeled NGAL test™ (BioPorto Diagnostics A/S, Gentofte, Denmark) on the Dimension Vista® 1500 system (Siemens Healthcare A/S, Ballerup, Denmark) using the Empty Flex Reagent cartridges® and following the application note.(Farnaud et al., 2010) Limit of detection was 15 ng/ml with measuring intervals of 25–3,000 ng/ml and 25–4,000 ng/ml. The analytical precision was estimated to 19.6%. The concentration was determined via the NGAL calibrator provided by the manufacturer. In case 13–21, saliva samples exceeding the measuring interval were diluted twice with isotonic saline and reanalyzed. This provided a tenfold higher measure interval (40,000 ng/ml). The manufacturer validated this method.

### 2.5 Amylase

The saliva and plasma amylase concentration were measured on the Dimension Vista 1,500® system (Siemens Healthcare A/S, Ballerup, Denmark) using the AMY method.

### 2.6 Microbial culture

Throat swab culture and pus culture were collected and transported using eSwab™. In brief, standard throat swab from patients with uncomplicated tonsillitis were cultured under aerobic conditions with CO2 on 5% blood agar plates (Herlev Hospital, Denmark). Throat swabs from patient with recurrent tonsillitis, peritonsillar abscess, or swabs containing pus were furthermore cultured under anaerobic conditions on agar plates containing Oxoid disks (Basingstoke, UK) with metronidazole 5 μg and kanamycin 1,000 μg as well as on chocolate blood agar plates (SSI Diagnostica, Hillerød, Denmark).

### 2.7 C‐reactive protein (CRP) and leucocytes

Plasma CRP was measured on the Dimension Vista® 1500 system (Siemens Healthcare A/S, Ballerup, Denmark) by a nephelometric method. Leucocytes in whole blood were measured on Sysmex hematology analytical module (XN‐20, Sysmex, Kobe, Japan).

### 2.8 Statistics

A calculated sample size of 15 cases was based on a population of 800,000 inhabitants, a confidence level of 95%, and a confidence interval on 25.(CR Systems, 2012)

The program R version 3.4.3 was used for statistical analyses. Statistical significance was set at *p* < .05. Students *t* test and Welch Two sample *t* test were used to test for comparison and linear regression when testing for correlation. A statistician performed the statistical analyses.

## 3 RESULTS

We included 21 cases: 12 females and 9 males (mean age 31, range 18–72 years), and 24 controls: 11 females and 13 males (mean age 40, range 18–72 years).

Most cases had an increased CRP and/or positive mouth swabs for bacteria. *Streptococcus pyogenes* was the most prevalent bacteria cultured. The most frequent diagnoses were acute tonsillitis (*n* = 10) and peritonsillar abscess (*n* = 6) (Table 1).

**TABLE 1:** Case data

ID	Sex	Age	Clinical diagnosis	NGAL‐saliva, pre mouth cleansing (ng/ml)	Neutrophil granulocytes (×10^9^/L)	CRP (mg/L)
1	M	19	Peritonsillar abscess	2,961	^†^	^†^
2	F	45	Parapharyngeal abscess	469	12.6	117
3	F	23	Acute tonsillitis	105	5.7	65
4	M	28	Acute tonsillitis	212	4.6	74
5	M	18	Acute tonsillitis	640	5.2	60
6	F	34	Pharyngitis without tonsillitis	600	15.0	15
7	F	65	Pharyngitis without tonsillitis	1941	18.2	200
8	F	21	Peritonsillar abscess	660	6.6	155
9	M	63	Acute tonsillitis & necrotizing fasciitis	>3,000^‡^	9.7	355
10	F	40	Peritonsillar abscess	1,035	11.5	68
11	F	67	Laryngitis	>4,000^‡^	16.3	188
12	M	18	Acute tonsillitis	821	3.1	34
13	F	25	Peritonsillar abscess	6,871	11.4	204
14	M	72	Parapharyngeal abscess	39,803	8.6	162
15	F	39	Acute tonsillitis	1870	12.2	65
16	F	37	Peritonsillar abscess	1,042	^†^	^†^
17	F	19	Peritonsillar abscess	766	7.6	92
18	M	18	Acute tonsillitis	1,606	5.7	143
19	M	19	Acute tonsillitis	854	9.3	158
20	M	18	Acute tonsillitis	3,569	17.8	258
21	F	33	Acute tonsillitis	1897	^†^	^†^

*Note*: ^†^missing value, ^‡^, concentration higher, but not known.

### 3.1 NGAL, saliva, in cases and controls, before mouth cleansing

**FIGURE 1:**
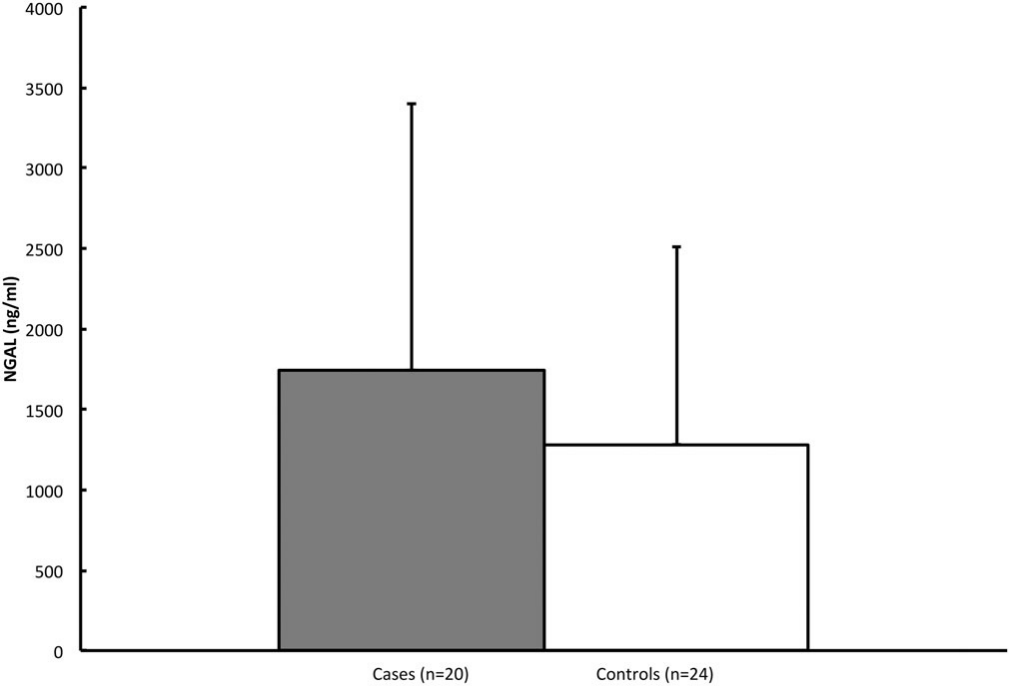
Saliva NGAL in cases with bacterial induced pharyngeal infection and controls. *The data are expressed as mean + *SD*

In both groups there was a great span in NGAL saliva concentrations. Case 14 presented an extreme NGAL saliva concentration of 39,803 ng/ml. This case was considered an outlier and excluded from all analyses. The mean NGAL concentration in cases was 1,746 ng/ml (105–4,000 ng/ml) (Table 1), and for controls 1,283 ng/ml (226–4,973 ng/ml). There was no significant difference in NGAL saliva concentration between cases and controls (*p* = .31) (Figure 1).

### 3.2 NGAL before and after mouth cleansing

**FIGURE 2:**
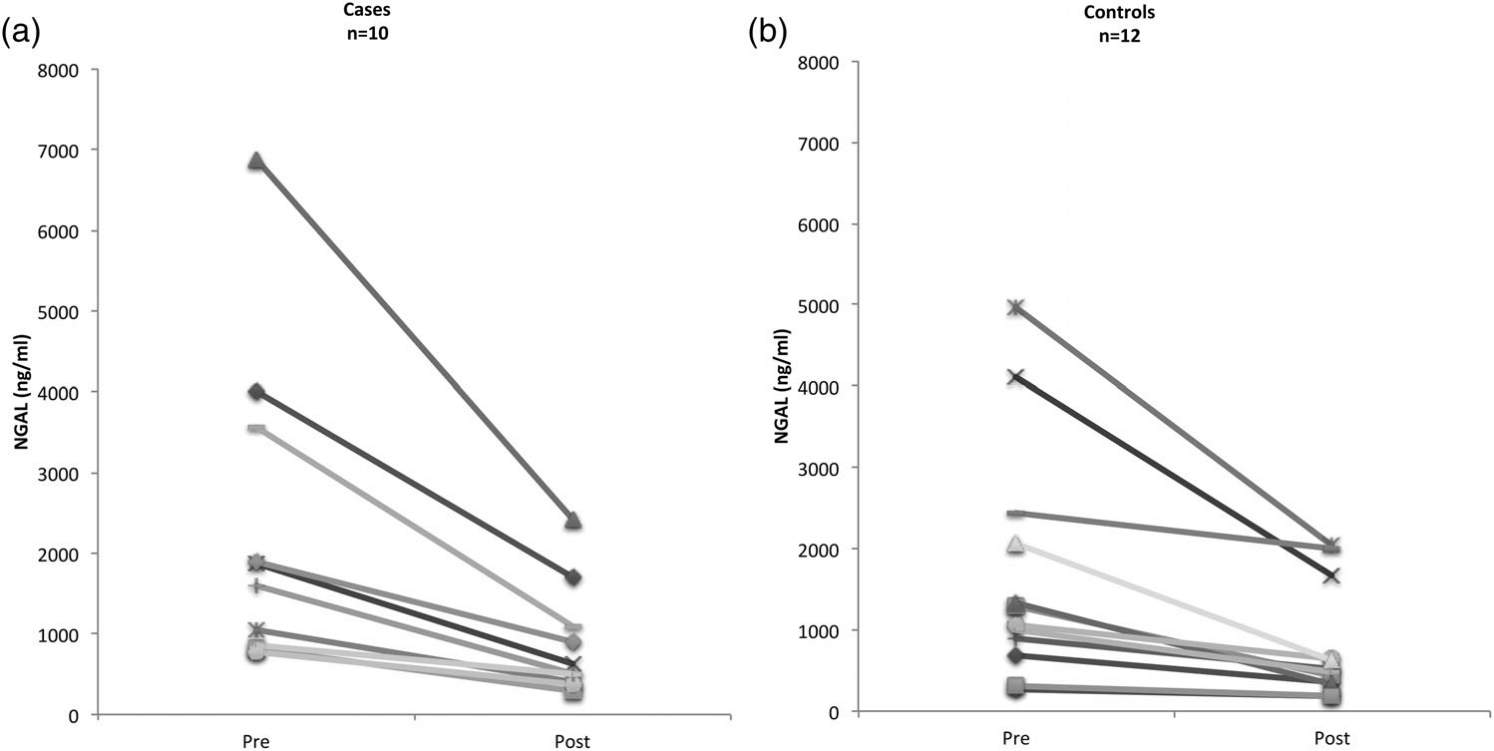
Saliva NGAL concentrations in cases with bacterial induced pharyngeal infections (a) and controls (b) collected pre and post mouth cleansing

The intra individual concentration of NGAL in saliva was compared before and after 5–10 s of mouth cleansing with tap water among cases (*n* = 10) and controls (*n* = 12) (Figure 2). For both cases and controls, the saliva NGAL concentration decreased significantly after mouth rinse (cases *p* = .01; controls *p* = .01), but there was no significant difference between the groups (*p* = .29).

### 3.3 NGAL saliva concentrations compared to plasma CRP and neutrophil granulocytes

**FIGURE 3:**
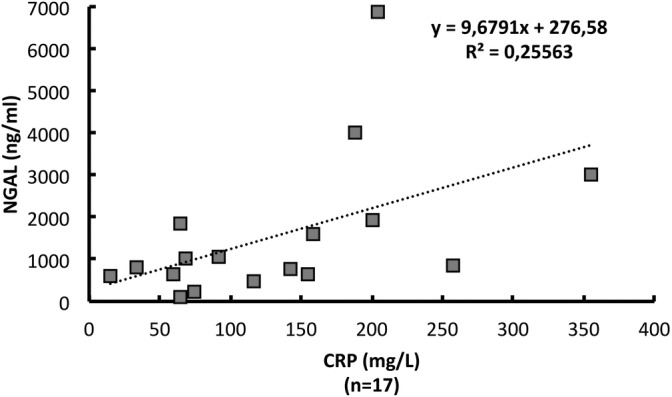
The correlation between plasma CRP and saliva NGAL in cases

Among cases, there was a significant positive association between saliva NGAL concentrations before mouth cleansing and plasma CRP concentrations (*p* = .001) (Figure 3). Whole blood neutrophil granulocyte count showed a nonsignificant positive correlation to saliva NGAL (*p* = .07).

### 3.4 NGAL saliva concentrations in relation to age and gender

There was no significant correlation between NGAL saliva concentration before mouth cleansing and age (cases, *p* = .38; controls, *p* = .78) or gender (*p* = .49).

### 3.5 NGAL saliva concentration compared to amylase saliva concentration

No significant relationship was found between salivary NGAL and amylase concentrations (cases, *p* = .86; controls, *p* = .44).

### 3.6 NGAL saliva concentration compared to plasma NGAL concentration

Plasma measures of NGAL were only obtained in cases, but no significant correlation could be demonstrated when compared to saliva NGAL concentrations (cases, *p* = .52).

## 4 DISCUSSION

There is an unmet need for rapid noninvasive diagnostic for pharyngeal infections, distinguishing between viral and bacterial infections. A test with this feature and purpose could potentially support decision‐making on initiation of antibiotic treatment. This pilot study examined if measuring salivary NGAL could meet this need. However, we could not demonstrate a significant difference in saliva NGAL levels between cases with acute bacterial throat infection and healthy controls. Nevertheless, this small pilot study has enlightened several potential issues that need to be considered in potential future hypothesis testing studies considering salivary NGAL as a biomarker.

NGAL fulfills several of the criteria needed for a good host response biomarker. First, the local biomarker should to some extend correlate with systemic markers. Indeed, we found a positive correlation between the local response, salivary NGAL concentration, and systemic host response, represented by plasma CRP. Additionally, an almost significant correlation between salivary NGAL (local) and blood neutrophil granulocyte counts was demonstrated. It is well known that blood neutrophil counts increase in response to systematic bacterial infections; but this is not always the case in local pharyngeal bacterial infections.(Bansal et al., 2014; Christensen et al., 2013; Koo & Eisenhut, 2011; Peltola et al., 2006)

NGAL serves as a defense/response molecule in tissues exposed to the outer environment, for example, the gastrointestinal canal, the airways, and the oral cavity.(Yang et al., 2002) The dimeric form of NGAL is mainly produced by neutrophil granulocytes in response to infection. Other cells like hepatocytes, renal tubule cells, endothelial cells, and macrophages also produce NGAL, but predominantly as the monomeric form.(Cai et al., 2010) The test essay used in this setting could not differentiate between the NGAL‐forms. Hence, the NGAL contractions measured could be the result of multiple simultaneous processes that complicates test result interpretation.

An oral bacterial infection biomarker should not be confounded by for example, age, gender, or poor oral hygiene. A previous study found that the saliva NGAL concentrations were higher in cases with periodontitis compared to healthy controls.(Morelli et al., 2014) In the present study, conducted by physicians and not odontologists, two cases (case 10 and 15) were evaluated to have a poor dental status and thus a potential poor oral hygiene.

A significant decrease in saliva NGAL concentration after mouth cleansing was measured in both cases and controls. Suggesting that identification of a reliable oral biomarker for pharyngeal infection is challenging, since multifactor confounders may influence the oral environment and the saliva composition. This could include factors like, gingivitis, periodontitis, inflammatory disease, salivary pH and viscosity, mucosal dryness, the composition and time since the last meal, to mention some.(Belstrom et al., 2020; Farnaud et al., 2010; Lynge Pedersen & Belstrom, 2019) Another way of achieving higher specificity could be repeated sampling. Oral mucosa has turnover rate of 4 hr and with proper cleansing this might make it possible to calculate NGAL delta values.(Dawes, 2003) This could at least in theory represent increased cellular turnover and release of NGAL suggestive of infection.

## 5 CONCLUSION

The present study could not demonstrate a simple association between the salivary NGAL concentration and pharyngeal bacterial infection. Furthermore, salivary NGAL concentrations were high among some controls, suggesting cofounders like periodontitis, not standardized salivary dilution level, and other exogenous factors increase the salivary NGAL level among controls, that is, healthy persons.

The NGAL molecular form most relevant for detection of an oral infection is not known, and the assay currently used does not differentiate between the forms. Before salivary NGAL can be used as a general oral bacterial infection biomarker, more information is needed regarding the oral cavity isoform of NGAL and from where it originates.

### 5.1 Limitations and further research

Being a pilot study, the case numbers are limited, which limits final conclusions. During the study we have acknowledged that further standardization of how and when to collect saliva samples is important. Before collection it is advisable to have a specific dental evaluation and periodontal diagnosis, identified and graded by odontologist according to standard staging systems. Second, it is learned from this study that for example, rinsing the mouth with tap water decreases markedly salivary NGAL levels.

Third, the measuring interval was initially set at 25–3,000 ng/ml and 25–4,000 ng/ml, respectively. Samples exceeding this interval were marked “>3,000” or “>4,000,” not specifying the precise NGAL saliva concentrations. Thus, potentially overlooking outliers or even neglecting a more significant difference between the two groups. However, we do not expect this limitation to change the main findings of this pilot study.

## CONFLICT OF INTEREST

The authors report no conflicts of interests.

## ETHICS APPROVAL

The study was approved by the local Danish Data Protection Agency, (NOH‐2017‐031, I‐Suite number: 06034). Individual informed and signed consent was obtained from each case.
